# Sexual dysfunction in women with breast cancer: The role of community midwives in early detection

**DOI:** 10.18332/ejm/156900

**Published:** 2022-12-22

**Authors:** Dimitrios Charos, Victoria Vivilaki

**Affiliations:** 1 Department of Midwifery, University of West Attica, Athens, Greece

**Keywords:** breast cancer, sexuality, sexual dysfunction

In recent years, many studies have shown that breast cancer causes significant changes in women’s sexuality. Breast cancer is a threat to female sexuality due to the symbolism of the breast in female sexuality, sexual pleasure, and femininity of women^1,2^.

Women who underwent mastectomy developed depression, body image disorder, and sexual dysfunction^2^, which negatively affected their self-perception^3^. The physical and emotional effects of cancer on women and their treatment affect their sexuality. Depression, pain, vaginal dryness and sleep disturbance are factors that contribute to the onset of sexual dysfunction^1,4^.

Women’s sexual dysfunction is directly related to body image and sexual expression, which are in turn affected by mastectomy and the side effects of their treatment (chemotherapy, radiotherapy and hormone therapy), not to mention the partner’s empathy with the emotional state of the woman^5-7^. Chemotherapy causes the highest rate of sexual dysfunction compared to other treatments^8^.

In younger women, sexual dysfunction is associated with chemotherapy and premature menopause^9^. Side effects from treatment combined with premature menopause, loss of sexuality and vaginal dryness are major problems for women^1^.

Similarly, a study shows that sexual problems stem from psychological factors, such as a negative body image, the feeling that they are not wanted by their partners, the perception of the couple’s relationship, etc^10^.

The main sexual dysfunctions^1,7,8,11,12^ that women experience after being diagnosed with breast cancer include: dyspareunia, vaginal dryness, decreased pleasure, fear of loss of fertility, negative body image, loss of femininity, loss of attractiveness, decreased sexual arousal, fatigue, decreased desire and sexual interest, lack of empathy in the intimacy of the couple’s relationship, anxiety, depression, etc.

In contrast, an interesting study showed opposite results. Women tended to also react quite differently with their sexual behavior; there were positive changes such as increased tenderness and affection. The frequency may be affected but not the quality of their sexual function^13^. In addition, women who underwent breast reconstruction had a better sex life, fewer depressive symptoms and a better body image^2^. The quality of a woman’s relationship may be an important predictor of sexual dysfunction^1,2,14^.

Difficulty in communication, lack of empathy by the partner, difficulty in resolving conflicts, lack of support from the partner, distress, lack of understanding and love, are signs of aggravation of sexual functions^14^.

Nevertheless, most women who experience sexual harassment find it difficult to seek care^12,15^, for fear of not finding the right one. However, a recent study reports that a large percentage of women and their partners have not received any information about sexuality^16^.

Women’s sexual health is not directly addressed by community midwives and other health professionals. Detecting and treating sexual dysfunction requires deeper understanding, communication and training of community midwives and other health professionals involved^1,6,17^.

Breast cancer may be a potential threat to female sexuality; however, community midwives and other health professionals do not seem to be sufficiently aware of the impact of breast cancer on women’s sexuality^1^.

Community midwives and other health professionals are often reluctant to inform women and their partners about sexuality after being diagnosed with cancer^16^. Community midwives need to be aware of the subjectivity of women’s sexual changes, as well as be properly trained on the topic^13^.

Midwives and other health professionals should evaluate, inform, and cultivate women’s sexual health in a climate of trust, intimacy, and active listening so that they can appreciate the women’s concerns and difficulties, in order to be able to suggest solutions and act accordingly^8,17^.

Community midwives in conjunction with an interdisciplinary team should identify the variables that affect sexual function and plan interventions^7^, as well as refer women to the appropriate professionals.

In addition to pharmaceutical and non-pharmaceutical solutions, improvement in sexual function has been observed in women undergoing cognitive-behavioral therapy (CBT)^6^. Psychoeducational interventions, enhanced communication with the woman, counseling for women and couples, group therapy as well as couple therapy, show a significant improvement in sexual mood and a positive body image^8,17,18^. Finally, midwife-based group education on sexual dysfunction and midwifery sexual counseling have been shown to improve women’s sexual health^19,20^.

Community midwives, who are close to women, need to be well-informed and well-trained on the sexuality of women with breast cancer. Usually, women are willing to report their sexual concerns in an environment in which they feel more comfortable.

## Data Availability

Data sharing is not applicable to this article as no new data were created.
